# Supplementary material for: Fine-scale appendage structure of the Cambrian trilobitomorph Naraoia spinosa and its ontogenetic and ecological implications
Source: Proc Biol Sci. 2019 Dec 4;286(1916):20192371. doi: 10.1098/rspb.2019.2371 (PMC6939273; doi:10.1098/rspb.2019.2371)
Supplement: Figures S1 and S2 [file rspb20192371supp1.doc]

**Supplementary Figures**

**Fine-scale appendage structure of the Cambrian trilobitomorph *Naraoia spinosa* and its ontogenetic and ecological implications**

Dayou Zhai, Gregory D. Edgecombe, Andrew D. Bond, Huijuan Mai, Xianguang Hou and Yu Liu


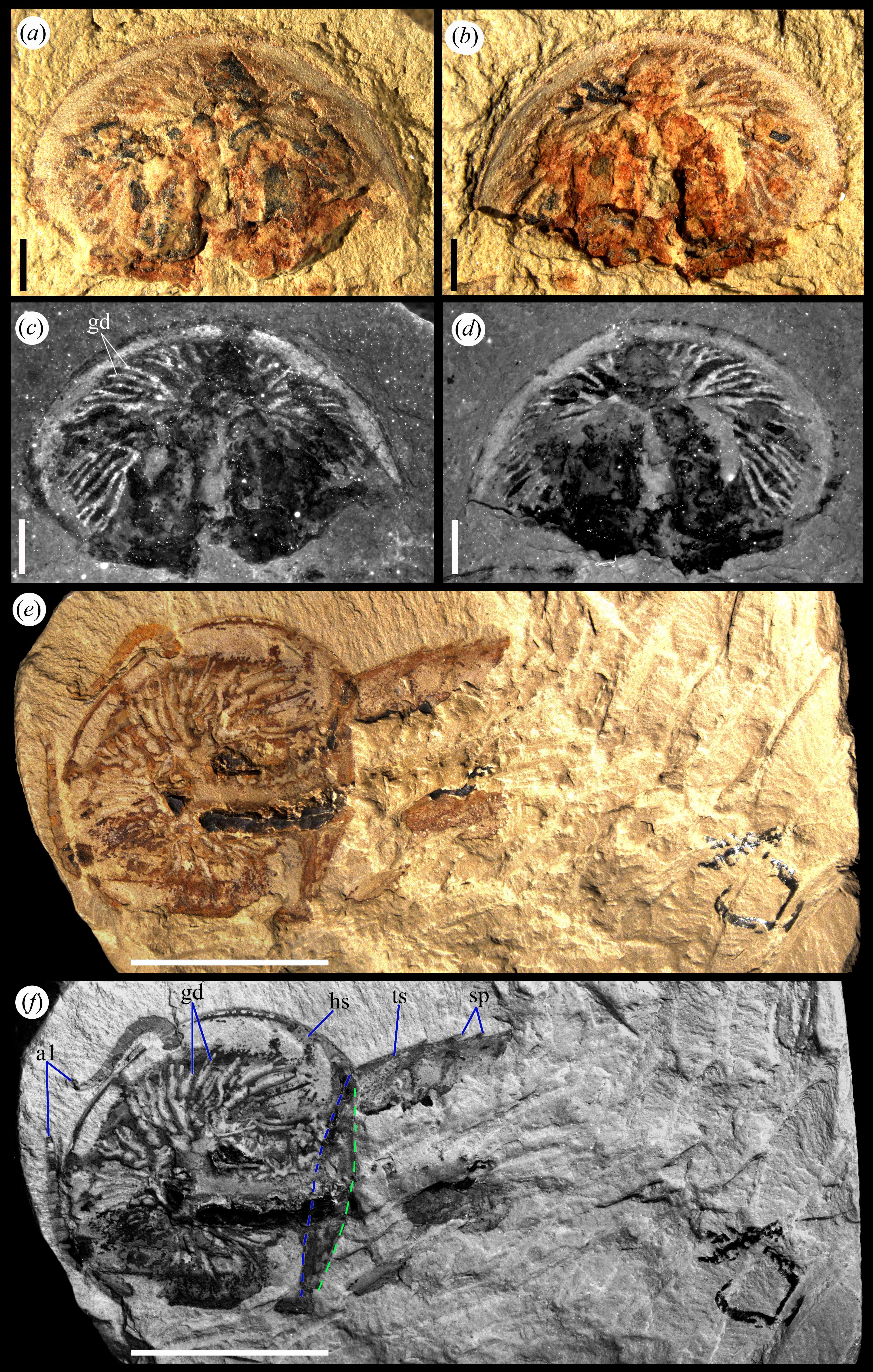


**Figure S1.** *Naraoia spinosa* Zhang & Hou, 1985, juvenile (*a*‒*d*, YKLP 11408) and adult (*e*, *f*, YKLP 11409) specimens. Related to figures 1 and 2. (*a*) Light microscopic photo of the part of juvenile, showing ventral view of the animal. The head shield is preserved mostly as imprints in this slab with most structures buried in the counterpart. (*b*) Light microscopic photo of the counterpart, showing dorsal view of the animal. (*c*) Fluorescence microscopic photo of (*a*). (*d*) Fluorescence microscopic photo of (*b*). (*e*). Light microscopic photo showing dorsal view of the adult. (*f*) Fluorescence microscopic photo of (*e*). Green dashed line represents posterior margin of head shield. Blue dashed line stands for anterior margin of trunk shield. Scale bars = 1 mm for (*a*‒*d*) and 5 mm for (*e*, *f*). Abbreviations: a1, antenna; gd, gut diverticula; hs, head shield; sp, spine; ts, trunk shield.


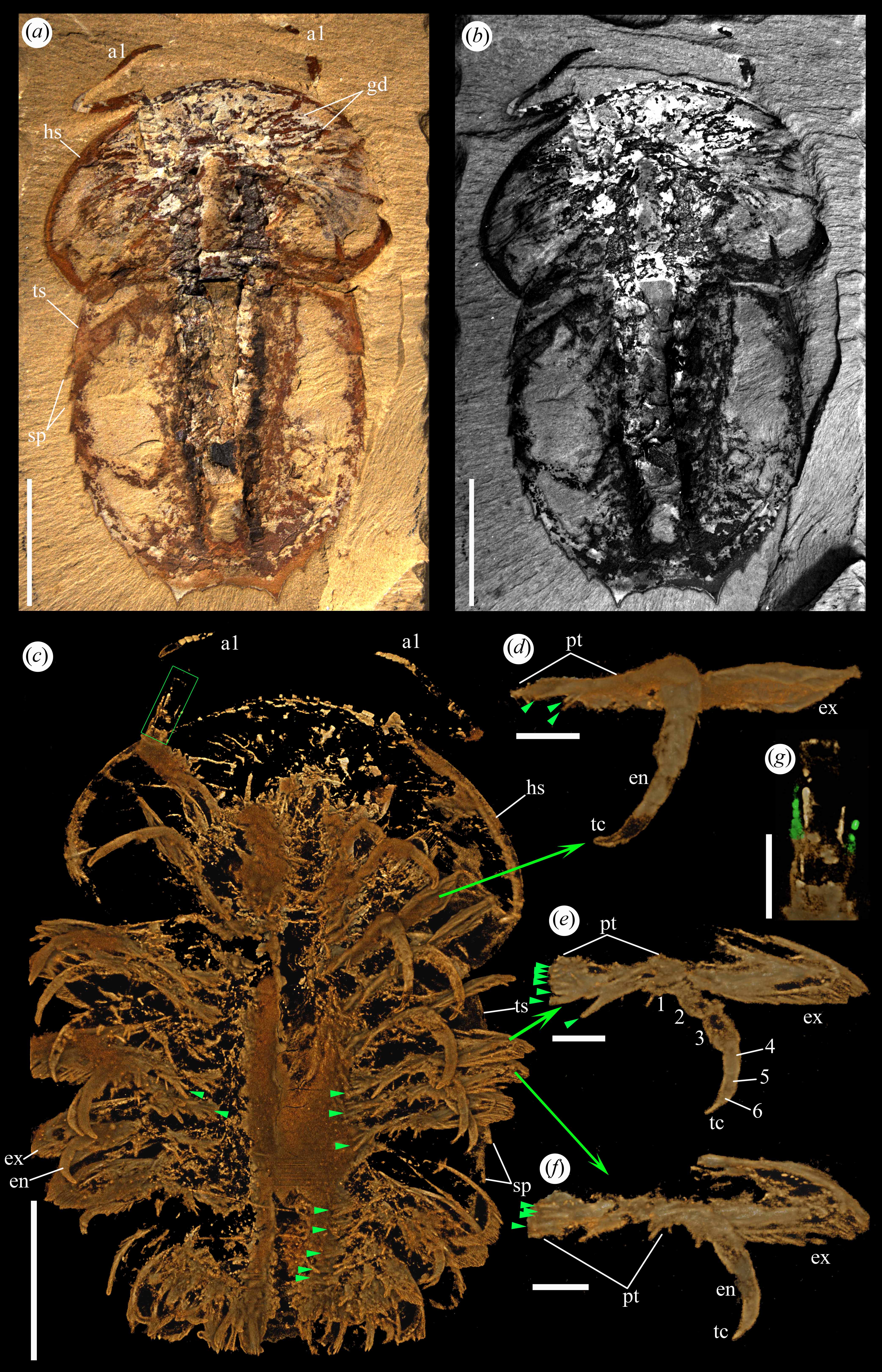


**Figure S2.** *Naraoia spinosa* Zhang & Hou, 1985, late-stage juvenile (YKLP 13941). Related to figures 1 and 2. Green arrowheads point to spines on protopods. (*a*) Light microscopic photo, showing dorsal view of the animal. (*b*) Fluorescence microscopic photo of (*a*). (*c*) Micro-CT image, showing ventral view of the animal. (*d*‒*f*) Dissected appendages from different parts of the body, interior views. (*g*) Enlargement of the green rectangle in (*c*). Green colouration showing apical setae on an antennal article. Scale bars = 5 mm for (*a*‒*c*) and 1 mm for (*d*‒*g*). Abbreviations as in figure S1. Numerals 1‒6 indicate endopodal podomeres.
